# Molecular analysis of photic inhibition of blood-feeding in *Anopheles gambiae*

**DOI:** 10.1186/1472-6793-8-23

**Published:** 2008-12-16

**Authors:** Suchismita Das, George Dimopoulos

**Affiliations:** 1 W. Harry Feinstone Department of Molecular Microbiology and Immunology, Bloomberg School of Public Health, Johns Hopkins University, 615N Wolfe Street, Baltimore, MD 21205-2179, USA

## Abstract

**Background:**

*Anopheles gambiae *mosquitoes exhibit an endophilic, nocturnal blood feeding behavior. Despite the importance of light as a regulator of malaria transmission, our knowledge on the molecular interactions between environmental cues, the circadian oscillators and the host seeking and feeding systems of the *Anopheles *mosquitoes is limited.

**Results:**

In the present study, we show that the blood feeding behavior of mosquitoes is under circadian control and can be modulated by light pulses, both in a clock dependent and in an independent manner. Short light pulses (~2–5 min) in the dark phase can inhibit the blood-feeding propensity of mosquitoes momentarily in a clock independent manner, while longer durations of light stimulation (~1–2 h) can induce a phase advance in blood-feeding propensity in a clock dependent manner. The temporary feeding inhibition after short light pulses may reflect a masking effect of light, an unknown mechanism which is known to superimpose on the true circadian regulation. Nonetheless, the shorter light pulses resulted in the differential regulation of a variety of genes including those implicated in the circadian control, suggesting that light induced masking effects also involve clock components. Light pulses (both short and long) also regulated genes implicated in feeding as well as different physiological processes like metabolism, transport, immunity and protease digestions. RNAi-mediated gene silencing assays of the light pulse regulated circadian factors *timeless*, *cryptochrome *and three *takeout *homologues significantly up-regulated the mosquito's blood-feeding propensity. In contrast, gene silencing of light pulse regulated olfactory factors down-regulated the mosquito's propensity to feed on blood.

**Conclusion:**

Our study show that the mosquito's feeding behavior is under circadian control. Long and short light pulses can induce inhibition of blood-feeding through circadian and unknown mechanisms, respectively, that involve the chemosensory system.

## Background

The circadian clocks, or oscillators, that influence a variety of activities of an organism are regulated by a range of external environmental factors, such as light, temperature, humidity, food and social interactions [1-5]. Genetic and molecular analyses in a range of evolutionary distant organisms, including the fruit fly *Drosophila melanogaster*, have revealed a striking degree of conservation of these oscillators and their underlying molecular mechanisms, many of which involve transcriptional feedback loops [6,7]. Timeless (tim) is one of the crucial factors that connects the endogenous clock with the external environment such as light [7]. When light pulses are applied early in the subjective night (soon after the lights have gone off) a phase delay is produced. Conversely, when light pulses are applied just before lights would have come on, a phase advance is produced [8]. Food is another potent clock-entraining cue, and the *D. melanogaster takeout *(*to*) gene has been shown to act as a molecular link between the circadian rhythm and feeding behavior [9,10].

Despite the essential role of circadian clocks in the regulation of vector behaviors that enable disease transmission, little is known about the molecular control of the host-seeking and feeding behavior of mosquitoes. It has been shown that the *A. gambiae *flight activity peaks during the night time and during the twilight [11]. Light has also been shown to inhibit *A. gambiae *flight activity and to re-set the rhythm by delaying it; however if the light phase begins early, it has a phase-advancing effect [12,13]. The host-seeking and feeding behaviors of many hematophagous insects exhibit rhythmic biting activity [14]; reaching peak levels in mid-night and are largely controlled by several circadian and chemosensory systems [15,16] involving a variety of odorant binding proteins (obp) and odorant receptors (or) that are linked to downstream signaling cascades [17,18]. In *D. melanogaster *antennae, the chemosensory components have been shown to be regulated by peripheral circadian oscillators [19].

Here we have initiated a detailed dissection of the delicate interactions that occur between the mosquito's light-sensing system, the circadian oscillators and the host seeking and feeding systems. Several studies have reported the influence of light on the flight activity of mosquitoes [11-13,20,21], while there is no comprehensive information available on the regulation of the mosquito's blood-feeding behavior by light, at least at the molecular level. We show that light pulses can induce changes in *A*. *gambiae *blood-feeding behavior and in the mosquito's global transcriptome, including transcripts that are regulated by blood feeding. We have functionally characterized selected genes that respond to the light stimulus by RNAi mediated gene silencing and have shown that they can moderately influence the mosquitoes' blood feeding behavior. We show that this modulation of the mosquitoes' blood feeding behavior is dependent on light pulse duration and occurs both through clock entrainment mechanisms and by clock independent masking effects [22-24] that may occur in a phase dependent or independent manner [25]. The light pulse stimulated alteration of mosquito feeding propensity implicates circadian factors that appear to down-regulate components of the chemosensory system.

## Results and discussion

### The blood feeding behavior of *A. gambiae *is under circadian control

**Figure 1:**
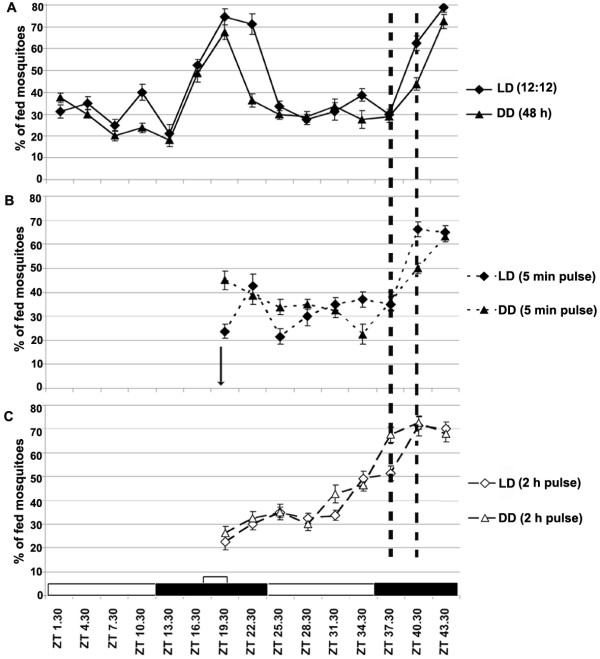
**The blood feeding behavior of *A. gambiae *is under circadian control**. A. A time course study of blood feeding propensity of mosquitoes maintained at normal conditions (LD 12:12) and at DD for 48 h. Blood was provided every 3 h, for 10 minutes and the percentage of mosquitoes that fed was plotted over time. The LD conditions of light on (ZT0) and light off (ZT12) are indicated with the white and black boxes, respectively at the bottom of the graph. The DD mosquito groups were maintained in darkness throughout the study. B. The blood feeding pattern for 24 h after mosquito was exposed to light pulses for 5 min (shown by an arrow) at 19.30 hours in both LD and DD conditions. C. The blood feeding pattern for 24 h, after mosquitoes were exposed to a 2 hr light pulse (shown by a white box) before 19.30 hours in both LD and DD conditions. Blood was provided every 3 h for 10 minutes and the percentage of mosquitoes that fed was plotted over time. The standard error bars are indicated in all the graphs.

To test whether *A. gambiae *blood feeding behavior is under circadian control we performed a time-course analysis of blood feeding propensity during a normal LD 12:12 light/dark cycle (12 h light, 12 h dark) and during a DD (dark-dark; continuous 48 hours) cycle to see whether the blood feeding propensity pattern would persist in absence of light (Figure 1A). We were interested in assessing the blood-feeding behavior of mosquitoes when kept in DD for 48 consecutive hours. In LD conditions, blood feeding propensity peaked during late night, at around 5–6 h after the light-off transition, and was low during the daytime. The persistence of this pattern even after a 48 h DD condition suggests that blood feeding propensity of mosquitoes is under circadian control. We also performed an experiment where mosquitoes were maintained in a DD condition for 6 days prior to feeding at a day and night time, to assess whether a free-run condition could be reached. These mosquitoes showed feeding behavior that did not correlate with the zeitgeber time-points (as in Figure 1A) and displayed a similar feeding propensity at both ZT19.30 and 28.30 hours, suggesting a free-run condition (data not shown); this finding further validates the circadian nature of blood-feeding behavior [26,27].

### Light pulse-induced alteration of *A. gambiae *blood-feeding behavior

A series of assays were performed to address the effect of long and short light pulses on the mosquito's blood feeding propensity. We investigated the effects of short (5 min) and long (2 h) light pulses, applied during the dark phase at 19.30 hours, on the blood-feeding pattern of mosquitoes in both LD and DD conditions (Figure 1B and 1C). A short light pulse of 5 min resulted in a transient inhibition (~30–50%) of blood feeding propensity in mosquitoes, in both LD and DD conditions (Figure 1B versus 1A). This observation suggests that the shorter light pulse did not evoke a behavioral phase shift, and the inhibition of feeding propensity is likely to reflect a masking effect [22]. On the contrary, a longer 2 h light pulse resulted in an immediate inhibition (~50%) of the blood feeding propensity along with a phase-advance (by ~3–4 h) in mosquitoes that were maintained at both the LD and DD conditions (for 48 h), compared to the non-light pulse stimulated mosquitoes (Figure 1C versus 1A). Earlier studies have shown that an early initiation of the light phase results in a phase-advancing effect on mosquito flight activity [12].

**Figure 2:**
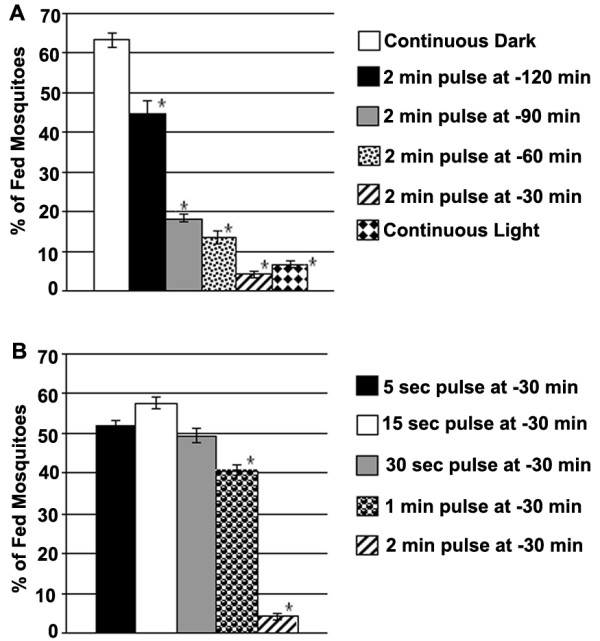
**Light pulse-induced alteration of *A. gambiae *blood-feeding behavior**. A. Mosquitoes were exposed to light pulses for 2 min at 120, 90, 60, and 30 min prior to the switch to the light phase and blood provision (ZT 0). Mosquitoes were allowed to feed for 10 min and the percentage of fed mosquitoes in each category was scored and plotted. Among the controls were one set that was not exposed to light (continuous dark) and another set that was exposed to continuous light for these 120 min prior to blood provision. B. Mosquitoes were exposed to light pulses for 5, 15, 30, 60 or 120 sec at 30 min prior to light onset and blood provision (ZT O) for 10 min, after which the percentage of fed mosquitoes were scored and plotted. Error bars indicate the standard error. Significant data points with respect to continuous darkness control set are marked with an asterisk.

We then performed a series of assays to better understand the mechanism mediating the inhibition of blood feeding propensity by shorter light pulses. Light pulses of equal duration (2 min) were applied at different durations (120, 90, 60 and 30 min) prior to blood provision and light onset (at ZT 0) (Figure 2A). In separate assays, light pulses of different duration (5, 15, 30, 60 and 120 secs) were applied at a constant duration (-30 min) prior to blood provision and light onset (at ZT 0) (Figure 2B). The first set of assays (Figure 2A) showed a negative correlation between the duration prior to feeding and the behavioral change as a measure of feeding propensity; the shorter the time between the light pulses and blood provision, the larger the decrease in feeding propensity. Mosquitoes maintained in constant light for 2 h prior to blood provision displayed the same degree of blood feeding inhibition to those exposed to a 2 min light pulse at 30 min prior to blood provision (less by ~55–60% compared to the continuous dark control) (Figure 2A). Interestingly, even a 2 min light pulse at 120 min prior to the light phase significantly decreased the mosquitoes' feeding propensity when compared to those not exposed to light. The second set of assays (Figure 2B) to examine the influence of the light pulse duration on feeding behavior showed that the light pulse duration was positively correlated with the induced behavioral change: the longer the light pulses, the larger the decrease in feeding propensity. A more detailed figure (non-fed, partially fed and fully fed mosquitoes) is presented in Additional file 1 and their corresponding values, standard error and the p-values (T-test) are shown in Additional file 2 . The light dosage and phase dependent fashion of blood feeding propensity modulation further supports the implication of a phase dependent masking effect rather than a phase advance when mosquitoes are subjected to the two minute short light pulses [22,23].

**Table 1:** Catalog of circadian (CIR) and chemosensory (CSR) genes that showed differential expression.

**Gene Name**	**Group**	**Transcript ID**	**Array "a"**	**Array "b"**	**Array "c"**	**Array "d"**	**Array "e"**	**Array "f"**	**Array "g"**	**Array "h"**
CK II BETA CHAIN	CIR	11438	**0.83**	0.56	0.51					-0.15
CCCP	CIR	01983			**-0.77**					0.56
CCCP	CIR	00750				**-1.44**				
TAKEOUT 1	CIR	04263			**-1.20**				-0.58	
TAKEOUT 2	CIR	12703			**-1.47**					**1.47**
TAKEOUT 3	CIR	04262			**-**0.59					**1.71**
TIMELESS	CIR	10787	**1.67**	**1.98**	0.36					
D7-RELATED 3 PRECURSOR	CSR	08283		-0.59	**2.33**				**1.95**	**-0.82**
GUSTATORY RECEPTOR	CSR	10195				**1.24**				-0.18
HORMONE BINDING	CSR	01352			**0.76**					
PHEROMONE BINDING	CSR	08054		-0.55	-0.49	**2.51**			**-1.07**	0.26
OBP	CSR	09388							0.39	**0.84**
OBP 15	CSR	03307			-0.43	**-0.79**		-0.73		
OBP 17	CSR	03309			0.49	**-0.97**				
OBP 19	CSR	04433			**-0.89**	**-0.89**	**-0.99**	**-0.81**		
OBP 22	CSR	10409			**-1.52**		**-1.49**			**0.76**
OBP 25	CSR	12320			**-0.91**					0.66
OBP 26	CSR	12321	**-0.78**		**-0.84**	**-2.31**	**-1.13**			-0.57
OBP 4	CSR	10489				**-1.58**				-0.36
OBP 47	CSR	07287			**-0.96**	**-1.36**	**-0.58**	**-0.27**		
OBP 49	CSR	06075							**2.14**	0.39
OBP 7	CSR	01556				**-1.16**				
PBP/GOBP	CSR	08279			**1.53**					-0.56
PBP/GOBP	CSR	08182							0.65	**0.84**
PHEROMONE BINDING	CSR	08055			**-0.75**	**-1.26**				

The table shows the list of CIR and CSR genes that were differentially regulated after either light pulse-treatment (arrays "a-f") or blood-feeding (arrays "g" and "h"). The log_2_-transformed fold-differential expression (if any) from the eight ("a" to "h") arrays are shown. The significant ones (above the threshold of ± 0.8; log_2 _1.75) are shown in bold. The transcript IDs have been shortened by deleting the first 5 alphabets "AGAP0" and only the last 5 characters are shown. [CK: Casein Kinase; CCCP: Circadian clock controlled precursor; OBP: Odorant binding protein; PBG/GOBP: Pheromone binding protein/general odorant binding protein].

For these blood feeding studies we chose an experimental design involving an artificial membrane feeding system (Parafilm M) with commercial human blood because of the difficulty in standardization and replication of parameters associated with the use of a mouse or a human arm, as it will be subjective to different individuals. Similar artificial membrane-feeding assays were also performed with close proximity (1–3 cm) to human hands and continuous human exhalations. These results showed an apparent insensitivity of the mosquitoes to the applied light pulse; the mosquitoes' feeding propensity was largely unaffected by light pulse after this chemosensory over-stimulation (data not shown). However this does not mean that the mosquitoes' feeding behavior upon direct stimulation with a human is not regulated by circadian oscillators that can be manipulated through environmental cues. The exposure of mosquitoes to a human arm or hand and exhalations at a 1- to 3 cm distance is an extreme condition providing maximal concentrations of chemosensory and heat stimulants, while in nature mosquitoes have to sense the human host at a distance of few hundreds to a couple of yards, at conditions that are very likely to be better simulated by a membrane feeding system that only allows for limited chemosensory stimulation [28].

It has been shown that the taste receptors in mosquitoes can be induced by phagostimulants such as adenine nucleotides [29]; thus the blood feeding behavior in mosquitoes is a complex process that is regulated by a variety of factors. Host seeking, and hence the blood-feeding propensity of mosquitoes, has also been shown to be stimulated by various cues emitted by the host, such as CO_2_, lactic acid, 1 octen-3-ol, heat and color [30]. In order to better understand the basis of the mosquitoes' attraction to the blood in the membrane feeding system, which may be regulated by the blood-specific odor, temperature and color, we designed a series of experiments that suggested that the mosquitoes were mainly attracted to a blood-specific chemosensory stimulant through the membrane feeding system (Additional file 3), and thereby supports the validity of our experimental design.

### Relationships between the light pulse and blood feeding – induced transcriptomes

To identify candidate components of the molecular circuits that mediate the interactions between the light stimulus and the mosquito's host seeking and feeding machinery, we used a microarray-based genome expression approach to assay differences in the mRNA abundance of genes under different light-stimulation (2 min and 2 h) and feeding conditions in whole mosquitoes and the head of both male and female mosquitoes (array-plan design in Additional file 4). An earlier observation that the *A. gambiae *host-seeking is inhibited for at least 24–36 h after a blood meal [31] was confirmed in our experimental system (data not shown) and was used as a basis for the selection of a 6-h post-blood-feeding time point for global gene expression analysis of blood feeding-induced gene regulation. A complete list of genes showing regulation after a light pulse and blood feeding are listed in Additional file 5.

**Figure 3:**
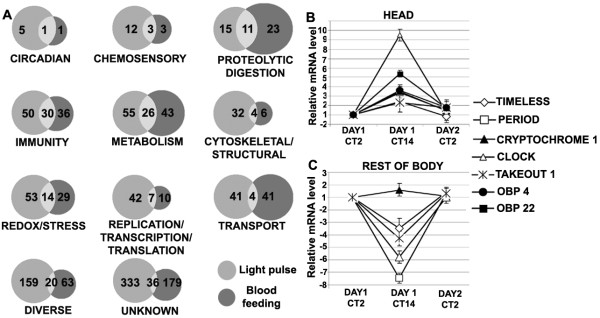
**Light pulse and blood feeding induced transcriptome**. A. Venn diagrams showing the number of genes regulated by light pulse (left, grey circle), blood feeding (right, black circles) and both stimuli (light grey shaded area in centre) in each of the different functional classifications: CIRCADIAN; CHEMOSENSORY; PROTEOLYTIC DIGESTION; IMMUNITY; METABOLISM; CYTOSKELETAL AND STRUCTURAL; REDOX/STRESS; REPLICATION/TRANSCRIPTION AND TRANSLATION; TRANSPORT; DIVERSE and UNKNOWN. The number of genes regulated in each sub-section is mentioned. The relative mRNA expression pattern of seven circadian/chemosensory genes at three different time-points of LD (12:12) conditions: in *A. gambiae *female head (B) and in the remaining body (C). The mRNA level at Day1CT2 was taken as 1 and the relative mRNA level at other time-points were calculated accordingly with respect to Day1CT2. [Day1CT2 = 2 h after light on stage on day 1; Day1CT14 = 2 h after light off stage on day 1; Day2CT2 = 2 h after light on stage on day 2].

A light pulse stimulation for 2 min at 30 min prior to RNA harvest (arrays "b" and "c"; see Additional file 4), resulted in the regulation of 345 genes in the total mosquito (274 up-regulated and 71 down-regulated) and 374 genes in the head (124 up-regulated and 250 down-regulated) of females (Additional file 4). Mosquitoes exposed to continuous light for 2 h (array "a"; Additional file 5) showed regulation of 188 genes (146 up-regulated and 42 down-regulated) in whole female mosquitoes. The regulated genes represented a variety of functional classes, of which the most abundant were metabolism, immunity, oxidoreductive processes, and transporters (Figure 3A and Additional file 4). Of particular interest were the genes with predicted functions related to the mosquito's circadian clock, chemosensory and blood-digestive systems (Table 1). The robustness of the microarray data was validated by qRT-PCR (Additional file 6).

#### Circadian clock-related genes

Light pulse regulated six circadian genes in the head or total mosquito: a *casein kinase *and the *tim *genes were up-regulated in the total mosquito, while the transcript abundance of two putative *to *homologs and two *circadian controlled precursor *genes were down regulated in the head (Table 1 and Additional file 5). Casein kinase and tim have previously been shown to be involved in regulating the circadian rhythm in response to light pulse stimulation in various organisms [7]. The mRNA abundance of the four major circadian genes: *period *(*per*), *clock *(*clk), cycle *(*cyc*) and *cryptochrome 1 *(*cry1*) was below threshold detection levels for microarray analyses (data not shown) at the assayed conditions (light on/off time-zone).

The *to *gene is involved in regulating the feeding behavior of *D. melanogaster *and has been shown to be up-regulated upon starvation [9]. A similar study in *A. gambiae *with sugar feeding has shown up-regulation upon sugar starvation (see later section). The down-regulation of two putative *to *transcripts (AGAP004263 and AGAP012703) by light pulse stimulation, which may account for the decreased feeding propensity (Figure 2), is intriguing when related to the RNAi gene silencing of *to *genes that results in an increased feeding propensity (see later section). This is likely to reflect other functions of these genes, as has been found in *D. melanogaster *[32]. A detailed phylogenetic study of the *A. gambiae to *genes with other homologues is discussed below. Two *to *transcripts (AGAP012703 and AGAP004262) were up-regulated in the carcass compartment after blood-feeding.

#### Chemosensory genes

As many as twelve genes with putative chemosensory functions were exclusively regulated by light pulse stimulation; three were exclusively regulated by blood feeding, and three by both stimuli (Figure 3A and Additional file 5 ). Nine *obps *were down-regulated after light pulse stimulation in the female head; this pattern is likely to be related to the lower blood-feeding propensity of the mosquitoes after light pulse stimulation (Figure 2). The *obp49 *gene showed significant up-regulation in female mosquito head after blood feeding; this increased activity may indicate a specialized function for this gene, such as mate seeking or oviposition site finding. Another interesting feature was the significantly lower expression of several *obps *(*15*, *17*, *19*, *26, 4, 47 *and *7*) in light pulse-stimulated female versus male heads, while a direct comparison between the gene expression in non-stimulated female and male heads showed a higher expression of three *obps *(*19*, *22 *and *26*) in the male heads. Female obps play major role in host seeking and blood feeding, while the male obps are involved in pheromone binding for mating and nectar feeding and may not be regulated by light-entrainable host-seeking behavior in the same way as the female genes. A specific obp can play multiple roles in sensing different types of complex odors, since the odor sensing is dependent on several obps that are part of a specific odor code [33].

A *D7 related 3 protein *which is distantly related to *obps*, was up-regulated in the head by both light pulse stimulation and blood feeding, and was down-regulated in the carcass after blood feeding (Table 1, Additional file 5). Proteins belonging to the D7 family has been shown to be expressed in the salivary glands and were injected into the host with saliva during blood feeding [34]. Among the other genes that showed regulation after light pulse stimulation includes a putative gustatory receptor, which was up-regulated in the female head when compared to the male head; and two pheromone binding proteins, of which one was up-regulated and the other was down-regulated in the female head (Additional file 5).

#### Blood digestive enzymes

As many as 23 genes involved in proteolytic digestion were regulated by blood feeding and 15 genes by light pulse stimulation, while 11 genes were regulated by both the stimuli (Figure 3A). The majority of these genes (17 out of 23) were up-regulated upon blood feeding; a finding that is consistent with the fact that this functional class plays a role in blood meal digestion. The *carboxypeptidase *genes were shown to follow a cyclical blood-inducible expression pattern with a peak every 3 h post blood meal [35], and in our assay two *carboxypeptidase *genes (ENSANGT00000016098 and AGAP011435) were up-regulated in the carcass after blood feeding, however AGAP011435 was down regulated in female head after a light pulse. Two *chymotrypsin *genes (chymotrypsin 1 and 2) have been shown to be induced in the midgut epithelium after blood feeding [36], and in our assay one *chymotrypsin *gene (AGAP010731) was similarly induced with feeding in female body, though it was down-regulated in female head after light pulse. In agreement with the down-regulation of two serine proteases in female body after blood feeding in our analysis, the gut-specific serine protease (AgChyL) has been earlier shown to be down-regulated during the peak hours of digestion (12 to 24 h) after a blood meal [37], although one of the serine protease (AGAP011477) showed up-regulation with light pulse in female head. An angiotensin converting enzyme and two aminopeptidase genes were down-regulated after blood feeding in the rest of the female body; however they were not regulated with light pulse. This finding is particularly interesting in light of a study that showed that *A. gambiae *aminopeptidase N (AgAPN1) was involved in blocking the development of *Plasmodium *ookinetes in several mosquito species [38].

#### Other functional gene groups

In *D. melanogaster*, a variety of physiological processes have been shown to be under circadian control and to be associated with periodic oscillations in gene expression patterns [39,40]. *A. gambiae *genes regulated by light pulse and blood feeding which are involved in other functional groups like immunity, oxidative stress and redox, cytoskeletal and structural, metabolism and other housekeeping genes (Figure 3A and Additional file 5) are discussed below. A larger number of genes were regulated by light pulse: immunity (50 genes), metabolism (55 genes), cytoskeletal and structural (32 genes), redox/stress (53 genes), RTT (42 genes) and TRP (41) functional groups, compared to those regulated by blood feeding (36 genes, 43 genes, 6 genes, 29 genes 10 and 41 genes respectively). In the "diverse" functional category, there were 159 genes that were regulated exclusively by light pulse stimulation, 63 exclusively by blood feeding and 20 by both stimuli. Of the genes whose functions were unknown, 333 genes were regulated exclusively by a light pulse, 179 exclusively after blood feeding and 36 by both stimuli (Figure 3A). A significant number of light pulse- and blood feeding-regulated genes were most likely not identified in these assays because of limitations of the microarray methodology to detect mRNA of lower abundance. These major changes in gene expression are indicative of the highly significant impact that a light pulse can have on mosquito physiology, at least partly by modulating circadian oscillators.

#### Immunity-related genes

As many as 50 genes with putative roles in the mosquito's innate immune system were found to be regulated by light pulse stimulation and 36 genes with blood-feeding exclusively. Thirty genes were regulated by both stimuli (Figure 3A) which adds up to 116 immune genes being regulated by the two stimuli (Additional file 4). These included genes encoding pattern recognition receptors (*fibrinogen binding protein *[*FBN*], *gram negative bacteria binding protein *[*GNBP*], *thioester containing proteins *[*TEP*], the *peptidoglycan recognition protein *family [*PGRP*], *c type lectin *[*CTL*], the *gal lectin *family [*GAL-E*], the *scavenger receptor *family, and the *leucine rich repeat protein *family [*LRR*]), serine protease cascade components (*CLIP domain serine proteases *and serpins), immune signaling pathway factors (*cactus*, *rel1 *and *rel2*), antimicrobial peptides (*cecropin*, *gambicin*, *lysozyme*, and *defensin*), and other functionally diverse proteins [41,42]. It was recently shown in *Drosophila *that disruption of the circadian rhythm is often correlated with increased susceptibility to infection [43]. The circadian regulation of the mosquito's innate immunity factors is particularly intriguing, since many of these molecules can modulate resistance to the malaria parasite *Plasmodium *[41]. Interestingly, blood feeding alone up-regulated several immunity genes such as *cecropin*, *fibrinogen immunolectins*, *leucine rich repeat protein*, *peroxidase *precursor, *thioester containing proteins *as well as *TOLL *precursors and *serpins*. Bacterial proliferation occurs in the gut after a blood meal and this is likely to result in immune gene induction [35,44]. Several immune genes were also down-regulated after blood-feeding and those includes among others; *c-type lectin 8*, *fibrinogen immunolectin 38*, a *scavenger receptor protein *and *spaetzle 4 *[35,45].

#### Oxidative stress and redox genes

A variety of oxidoreductive and stress-related genes were found to be regulated upon light pulse stimulation (Figure 3A). Genes in this group included those encoding *cytochromes*, *glutathione S-transferases *(*GST*s), *oxidoreductases*, *dehydrogenases *and *heat shock proteins*. Most of the detoxification-related genes (18 genes); such as the *cytochrome P450*, *GST *and *DNAJ *genes, were down-regulated after light pulse stimulation in the female whole mosquito and head. These genes have been linked to insecticide resistance [46]. Light pulse stimulation can change the redox state by generating several ions/molecules, resulting in up-regulation of several of these genes, including those for oxidoreductases, mitochondrial carrier proteins, heat shock proteins, and several dehydrogenases [39]. It has been shown earlier that several detoxification enzymes are up-regulated after blood feeding, as a result of the release of reactive oxygen species during the digestion of heme [45]; a similar pattern observed in our assay, with most enzymes being up-regulated after blood feeding.

#### Cytoskeletal and structural genes

Thirty two genes encoding components of the cytoskeleton and those involved in structural maintenance, such as those encoding *actin*, *myosin*, *tubulin*, *kinesin*, *tropomyosin*, *cuticle proteins *and *dynein*, were mainly up-regulated (with few exceptions) by light pulse stimulation in both head and whole mosquitoes (Figure 3A). Six genes were regulated with blood-feeding exclusively and 4 genes were regulated by both stimuli (Figure 3A); hence 42 genes were regulated by both stimuli (Additional file 4). Many of these genes have previously been shown to be differentially expressed after blood feeding [45], and some play a role in the *Plasmodium *parasite's traversal of the midgut epithelial cells and the repair of the midgut epithelium after this invasion [42]. Of particular interest are the *peritrophin precursor *gene, which is up-regulated in female head after both light pulse stimulation and blood feeding, and an *annexin *gene which was down-regulated in female head after light pulse stimulation. An annexin protein has been previously shown to be up-regulated by light pulse stimulation and serotonin in the eye circadian system of *Aplysia *species [47].

#### Metabolism and other housekeeping genes

As many as 55 genes encoding a variety of enzymes involved in a range of different metabolic pathways were differentially regulated upon light pulse stimulation; 33 were up-regulated and the remaining 22 were down-regulated in both whole female mosquitoes and in their heads (Figure 3A). A variety of replication-, transcription-, and translation-related genes (42 genes) were regulated by light pulse stimulation and to a lesser extent after blood feeding (10 genes), in both the head and whole mosquito Additional file 5. A variety of metabolic processes are tightly controlled by circadian oscillators, since these processes need to exhibit different activity levels during the day and night phases [39,48]. Genes related to transport processes (41 genes) were also regulated (mainly up regulated in total mosquitoes) with light stimuli; this finding is consistent with reports that light entrainment is associated with adjustments in several photoreceptor molecules, and several ions, solute factors, and bio-molecules may need to be transported to other cellular compartments [39].

### Circadian expression of the clock and chemosensory genes in *A. gambiae*

The regulation of several clock and chemosensory genes by a short light pulse stimulation (Figure 3A) which, however, was not capable of inducing a phase advance was intriguing (Figure 1B), and we pursued further characterization of selected genes (*tim*, *per*, *cry 1*, *clk*, putative *to 1*, obp4 and obp22) with regard to their mRNA abundance at successive time points over a LD 12:12 cycle to test for their circadian regulation (Figure 3B and 3C). This analysis showed some interesting features that may suggest that the circadian system of hematophagous insects share certain commonalities. All seven genes showed a higher expression pattern in female head at the night-dark phase (CT14), similarly to what has been observed in *D. melanogaster *[39]. The expression pattern of these genes (except *cry 1*) showed a quite different expression pattern in the rest of the body where their mRNA abundance displayed the lowest level during the night-dark phase (CT14). A similar observation has been shown for the blood sucking sandfly *Lutzomyia longipalpis*, where several clock regulated genes were regulated in an opposite manner in female head and body [49].

### Determination of RNAi knockdown efficiency and expression analysis of selected circadian and chemosensory genes

To identify putative components of the molecular interactions between the mosquito's light sensing system, light pulse-entrained circadian oscillators and its host-seeking and feeding systems, we selected 10 genes for RNAi gene silencing assays in adult female mosquitoes to assess their potential influence on the mosquito's blood-feeding behavior. Seven of these genes (*tim*, putative *to1*, *to 2*, *to3*, *obp4*, *obp22*, and *obp26*) were selected on the basis of their expression patterns (Additional file 5), and the remaining three (*per*, *clk1 *and *cry*) were included as representative of previously identified circadian clock factors. A series of assays were first carried out to determine the silencing efficiencies tissue specific expression specificities of the targeted genes.

**Figure 4:**
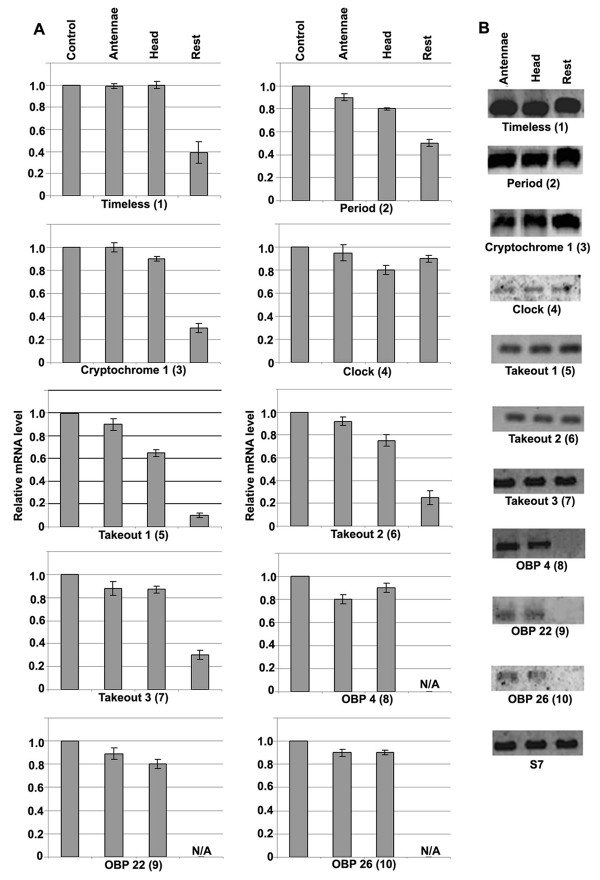
**Determination of knockdown efficiency and the tissue specific expression patterns of circadian and chemosensory genes**. A. Determination of knockdown efficiency (by qRT-PCR; 4 days after dsRNA injections) of 10 genes (*per*, *tim*, *clk*, *cry 1*, putative *to1*, *to2*, *to3*, *obp 4*, *obp 22*, and *obp26*) in antennae (that includes maxillary palps and proboscis), head, and the rest of the body of *A. gambiae *adult female mosquitoes. The level of the gene transcripts in each body part was normalized with AgS7. The graph shows the relative expression of each gene after knockdown, as compared to that for the GFP treated control mosquitoes (set to 1.0). N/A refers to the tissue in which the corresponding gene is not expressed. The error bars indicate the standard errors. B. Tissue specific expression (qRT-PCR products run in agarose gel) of the above mentioned 10 genes in adult female mosquitoes: the antennae (including maxillary palps and proboscis), head and the rest of the body. For all three body parts, equal amounts of RNA were taken and S7 gene of *A. gambiae *was the internal control. The numbers in brackets corresponds to the 10 genes in both A and B sets.

RNAi-mediated gene silencing assays in adult mosquitoes depend on direct injection of dsRNAs into the hemolymph, which resides in the insect's open circulatory system. The dsRNAs are then taken up by various tissues and cell types. It is reasonable to expect that some tissues, such as the antennae, are less accessible to this dsRNA and would be less susceptible to RNAi-mediated gene silencing because it is more difficult for the double-stranded RNA to diffuse into these body parts from the thoracic hemolymph. Some of these genes are known to be expressed in specific tissues, where they are most likely regulated by peripheral circadian oscillators; others would be expected to display a more general mRNA abundance in multiple tissues. For instance, the obps are known to be mainly localized in the antennae, proboscis, maxillary palps and head [50,51]. The RNAi-mediated gene-silencing efficiency was highest in the remaining body parts for the majority of the tested genes (ranging from 90% for putative *to1 *to 20% for *clk*), while the head and antennae displayed lower silencing efficiencies (10 – 20%) (Figure 4A and Additional file 7). However, even a relatively low gene-silencing efficiency, as determined by a decreased abundance of mRNA, may still produce a significantly altered gene knockodown phenotype, as has been shown previously in *A. gambiae *[52].

The transcript abundance of these genes was assessed in the antennae (including proboscis and maxillary palps), the head, and the remaining body of 4-day-old female mosquitoes (Figure 4B). Since the circadian clock components have a rhythmic cyclical expression, RNA samples were collected at 4 hr intervals over a 24-h period (at CT0, 4, 8, 12, 16, 20 and 24) and then pooled for analysis of tissue-specific expression. The *tim and per *transcripts and all three putative *to *transcripts were detected at equal levels in all body parts, while *cry 1 *and *clk *showed slightly lower expression in the antennae than in the other body parts (Figure 4B). The three *obps obp4*, *obp22*, and *obp26 *were expressed only in the head and antennae and not in the rest of the body, consistent with previous reports [50,51,53].

### Modulation of blood-feeding behavior through gene silencing of light-pulse regulated circadian and chemosensory factors

**Figure 5:**
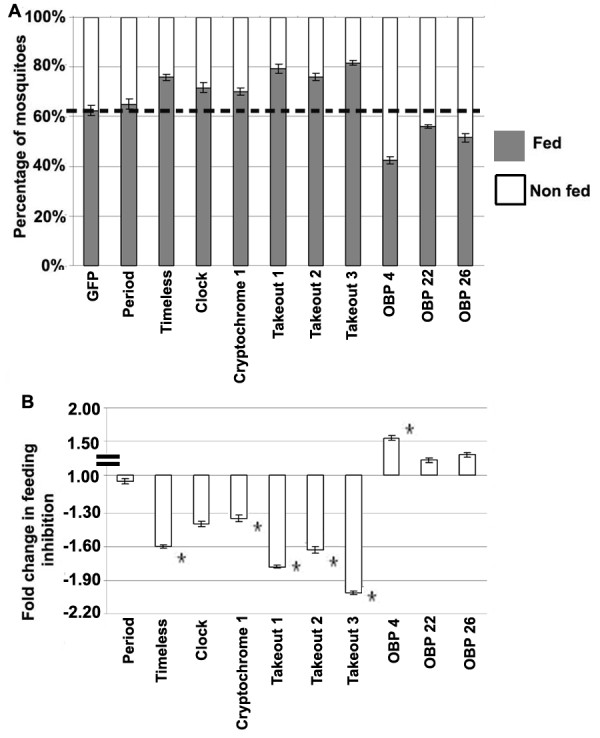
**The effect of gene silencing on blood-feeding propensity of adult female mosquitoes**. A. The percentage of gene silenced mosquitoes that fed (black bar) and that did not feed (white bar) upon blood provision after injection of dsRNA. The difference between the broken line (corresponding to GFP control) and the top end of the bar for the fed mosquitoes of each silenced genes represents the percentage change in feeding propensity compared to the GFP control. B. The fold change in feeding inhibition (non-fed) of the gene silenced mosquitoes with respect to the GFP dsRNA treated control mosquitoes. The standard error for three replicate assays is indicated with bars. The significant ones are marked with an asterisk.

We used an RNAi-based gene silencing approach to test some of the light pulse regulated genes for implication in the molecular interactions between the mosquito's light cue sensing systems and its host-seeking and feeding systems. The feeding propensity of the *tim*, *clk*, *cry 1 *and putative *to *gene-silenced mosquitoes was increased by ~10 – 20%, whereas silencing of *obp4 *decreased the feeding propensity by ~20%, and silencing of *per*, *obp22*, or *obp26 *had no apparent effect on the feeding phenotype when compared to the GFP dsRNA-treated control mosquitoes (Figure 5A and Additional file 8). The statistical analysis (T-test and Mann Whitney [41,54]) showed that *tim*, *clk*, *cry 1*, three putative *to *and the *obp4 *gene-silenced mosquitoes had a significantly increased feeding propensity (Additional file 8) compared to the GFP dsRNA treated control mosquitoes. The mRNA abundance of all ten circadian genes used for RNAi gene silencing assay are shown at some of the light on/off time-zone assayed conditions by both microarray and qRT-PCR assay (Additional file 9). The increased feeding propensity in *tim-cry1*- and *clk*-silenced mosquitoes may be explained by an effect on the circadian clock, causing a delay in reaching the light-onset phase when feeding propensity is normally low (Figure 1A). The increased blood feeding propensity in *to *silenced mosquitoes suggest that these genes influence feeding behavior in the mosquito, similarly to what has been observed in the fruit fly [9]. The decreased blood-feeding propensity of *obp4*-silenced mosquitoes supports a role for this chemosensory factor in host seeking. The low level of feeding inhibition that was produced by depletion of two other *obps *(*obp22 *and *26*) is likely a result of the inefficient RNAi silencing of these genes in the antennae. The fold difference in feeding inhibition (% non-fed of gene silenced divided by % non-fed of GFP) is shown in Figure 5B.

### Phylogenetic analysis of the takeout homologs of *A. gambiae *and their regulation in response to starvation

**Figure 6:**
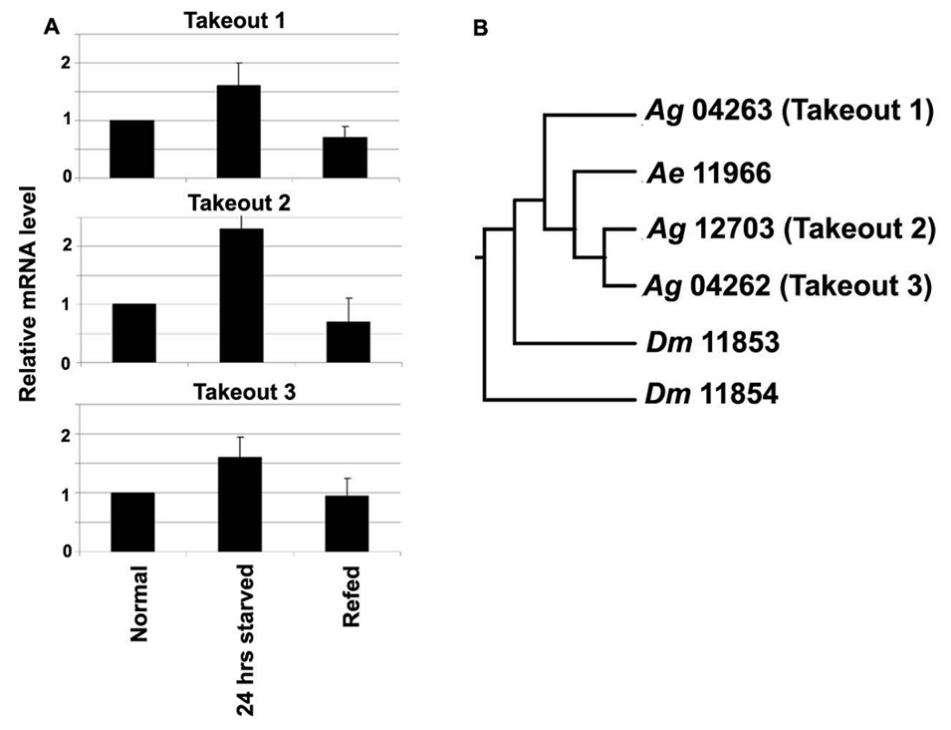
**The Takeout family**. A. *Takeout *gene expression (*to1*, *to2 *and *to3*) in response to sugar starvation and re-feeding. Expression was determined by qRT-PCR in 4-day-old adult female mosquitoes (normal) and 24-h sugar-starved and sugar re-fed (after 24-h starvation) mosquitoes. The standard error for three replicate assays is indicated with bars. B. Dendogram of three putative takeout proteins of *A. gambiae *(Ag04263, Ag12703, and Ag04262; with the last 5 numbers of the ENSEMBL AGAP IDs), *A. aegypti *takeout protein (Ae11966; the last 5 numbers of the VectorBase gene ID number), and two *D. melanogaster *takeout proteins (Dm11853 and Dm11854; the last 5 numbers of the Genbank accession number). The full gene IDs are mentioned in the main text.

The *D. melanogaster to *transcript has been shown to be induced by starvation and been proposed to participate in a novel circadian output pathway that translates temporal and food status-related information into feeding-relevant metabolism and activity [9,10]. Two *A. gambiae takeout *genes (AGAP012703 and AGAP004263) identified earlier in the antennae, [55] and one *Aedes aegypti to *gene (AAEL011966) have been shown to be orthologs of the *D. melanogaster *takeout protein [56]. In a separate study, based on sequence similarity searches using the *Aedes *takeout protein sequence (AAEL011966), another 25 *Drosophila *and 13 *Anopheles *homologs have been identified [50]. Of these, only two *Drosophila *proteins (CG11853, CG11854) and one *Anopheles *protein (AGAP004263; AgTOL-2) displayed all the conserved features of a takeout protein; the others contained only the conserved secretory juvenile hormone binding protein (JHBP) domain. We used the *A. gambiae *putative takeout (AGAP004263; Ag-TOl-2) protein sequence in BLAST searches to identify two additional members, AGAP012703 and AGAP004262, both of which also showed differential regulation by light pulse and blood feeding in the microarray analysis (Table 1). We have designated the three genes AGAP004263, AGAP012703, and AGAP004262 as putative *takeout1*, *takeout *2, and *takeout3*, respectively. The three *A. gambiae *putative *to *genes were analyzed in terms of their potential sugar starvation-dependent regulation and showed a 1.5- to 2.5-fold up-regulation after a 24-h sugar starvation (Figure 6A). These transcripts returned to the pre-starvation levels of abundance after a 10% sugar solution was provided; as previously shown with re-feeding in *D. melanogaster *[9]. Phylogenetic analysis of the six insect protein sequences (three from *A. gambiae*, two from *D. melanogaster*, and one from *A. aegypti*) indicated that *A. gambiae *AGAP004263 is more closely related to *Aedes *AAEL011966 and *Drosophila *takeout proteins (CG11853, CG11854) than are the other two *A. gambiae *takeout proteins (AGAP012703 and AGAP004262) (Figure 6B). The down-regulation of three putative *to *genes in the head and up-regulation in the rest of the body with blood feeding (Additional file 5), further support differential functions of these factors in different parts of the mosquito in response to assorted kind of feeding (sugar versus blood).

## Conclusion

Our study has established an important role for the light modulated circadian and chemosensory oscillators in the regulation of the mosquito's blood feeding behavior, and consequently its capacity to transmit blood-borne pathogens. While previous studies have described the *A. gambiae *flight activity during the night time and during the twilight [11,12], here we provide insight into the molecular components and mechanisms that regulate the blood feeding behavior. We have shown that the mosquito's blood feeding behavior is under circadian control, with a peak during the late night (Figure 1). A short 2 minute light pulses can momentarily decrease the mosquito's blood-feeding propensity in a non-circadian fashion, while a longer light pulse of 2 hours can induce a phase advance phenomenon in the mosquito's feeding behavior, similarly to what has been shown for the circadian regulation on flight activity [12]. The short 2-min light pulse can cause a profound change in the mosquito's transcriptome that reflected a modulation of a variety of physiological systems, including those that regulate the mosquito's vectorial capacity. We have mainly focused on transcripts that influence mosquito's feeding behavior; future studies are needed to more clearly define the specific ways in which light can contribute to the regulation of the mosquito's anti-*Plasmodium *defense system and its resistance to infection. The overlap between the light pulse- and blood feeding-induced transcriptomes encompassed a variety of genes, some of which represent molecular links between the mosquito's light sensing and feeding systems. The dosage dependent effect of the short 2-minute light pulse on feeding propensity (Figure 2) over time suggests it may represent a masking effect which however involves several clock factors (Figure 3A). Further detailed studies are required to better understand the impact of a masking mechanism on the mosquito's blood-feeding behavior, and whether this is a phase dependent or independent system. The circadian expression pattern and down-regulation of several *obp *transcripts after light pulse stimulation (Table 1), in conjunction with the gene-silencing phenotypes observed for these genes (Figure 5), establishes them as essential factors in the mosquito's host-seeking system and further supports an important role of the mosquito's chemosensory oscillators in the regulation of host seeking and feeding behavior [15,16,33]. Future work should focus on the characterization of these links since a detailed understanding of these interactions can lead to the development of novel malaria control strategies based on interference with the mosquito's host-seeking behavior.

## Methods

### Rearing of *A. gambiae *mosquitoes

*A. gambiae *Keele strain mosquitoes were raised at 27°C and 70% humidity, and adults were maintained on a 10% sucrose solution according to standard rearing condition [57]. The insectary room was equipped with a Philips 25-watt T8 fluorescent lamp (3500K; daylight spectrum) having an intensity of ~800–1000 lux.

### The blood feeding behavior of *A. gambiae *adult mosquitoes and the light pulse treatments

Each type of blood-feeding assay was repeated at least three independent times; with three replicas (of ~20 mosquitoes each) every time. In all assays, the 37°C blood meal was provided by standard artificial membrane feeding system (Parafilm M) for 10 min. The females were first entrained to the 12-h LD cycle of the insectary for 5–6 days before any assays were carried out.

For the time course study of mosquito blood feeding behavior; three mosquito cups (for 3 replicas) were given 37°C blood meal for 10 min, every 3 h (Figure 1). The set that was kept in constant DD, three cups for each set were kept in airtight plastic containers covered with aluminum foil for 48 h before the experiment was conducted. Every 3 h during the assay, one box was opened and the mosquitoes were given 37°C blood meal in dark for 10 min. For mosquitoes that were given light pulses (Figure 1B and 1C), mosquito cups for each set were kept in separate airtight transparent plastic container (3 replicas for each) and maintained in DD or LD conditions. At or before 19.30 hours, light pulses were given (for 5 min or 2 h), after which blood meal was provided for the next 24 hours, every 3 h. The DD or LD conditions were maintained even during blood-feeding time for all the assays.

For the light pulse assays in early morning hours (Figure 2); the night before the assay, each plastic container (containing 3 mosquito cups) was covered with aluminum foil just as the light went off for the night (light intensity in dark was ~1.5 lux). The following day prior to light phase initiation, light pulses were given at different time points and for different durations to each set (Figure 2A and 2B); by removing the aluminum foil and the cover of the respective boxes. After the light pulses, the boxes were covered with aluminum foil and returned to their original room, which was still in dark. The mosquitoes were given a 37°C blood meal for 10 min, when the light went on in the insectary (ZT 0). After feeding, the mosquitoes were sorted according to the amount of blood they had ingested: those that had not fed at all (non-fed), those with a fully stretched abdomen (fully fed) and those with a small quantity of blood in the abdomen (partially fed) (Figure 2 and Additional file 1). Statistical analysis Students T-tests were done and the p-values are shown in Additional file 2.

In all the assays, care was taken not to disturb the mosquitoes and to minimize their exposure to other external stimuli during the light pulse treatments: the experimenter wore a lab coat, double gloves, and a face mask. The light pulses were given to each set in a different room, so as to avoid the unnecessary exposure of any other experimental boxes to the light. In addition, all the different mosquito groups were always contained in their respective airtight plastic containers to reduce the exposure of other mosquito sets when working with one particular set.

### RNA isolation and quantitative real-time PCR (qRT-PCR)

RNA was extracted and quantified (in triplicate) either from whole mosquitoes or separately from the antennae (also including the maxillary palps and proboscis), the head, and the rest of the body, using an RNeasy kit (Qiagen, Valencia, California, USA). The qRT-PCR assays were performed as previously described [41] and the ribosomal protein S7 gene was used for normalization of the cDNA templates. The fold differences in expression levels or gene silencing efficiency (see below) were calculated according to the standard E^ΔΔCt ^method [58].

### RNAi gene-silencing assays in adult mosquitoes

RNA interference (RNAi) assays in adult female mosquitoes were performed using established RNAi methodology [59] injecting ~200 ngm dsRNA per mosquito. To silence the *obp *genes in head and antennae; at least 7–8 times more dsRNA (~1400–1600 ngm per mosquito) were injected to get a better silencing efficiency in these body parts, where diffusion of dsRNA through hemolymph would be less accessible. Three independent RNAi gene-silencing assays were performed for each gene with two replicas each time (~30 mosquitoes in each replica). The sequences of the primers used for making dsRNA are provided in Additional file 10. The gene silencing was verified by qRT-PCR, 4 days after injection of the dsRNA, and the *A. gambiae *ribosomal protein S7 gene was used for normalization of the cDNA templates [58]. The primers used for silencing verification are presented in Additional file 11. To study the blood-feeding behavior of the mosquitoes after RNAi gene silencing; 4 days after dsRNA injections the mosquitoes were blood-fed at the time of light onset in the insectary (described earlier) and were scored for blood-fed mosquitoes versus unfed. For statistical analysis, Students T-tests and Mann Whitney tests were done and the p-values are shown in Additional file 8.

### Microarray analysis

RNA was extracted (in triplicates) from total or head of the mosquitoes (as described earlier) of light pulsed and blood fed mosquitoes. The control samples were non-pulsed and unfed mosquitoes respectively. Sample preparations and microarray hybridizations were performed as previously described [41] with the experimental samples being labeled with Cy-3 and control samples being Cy-5 labeled. Scanning and data analyses were performed as previously described with a signal cut-off intensity of 100 to remove low-intensity/poorly hybridized spots from the analysis. Loc-Fit normalization (LOWESS) was performed independently for each data set through MIDAS (available online, TIGR). The TMEV software (available online, TIGR) was then used for SAM analysis with a 5% false discovery rate (FDR). Only transcripts that had signal values above or below the log_2 _cutoff value of ± 0.80 were used for further analysis as previously described. The expression data generated by the microarray were validated independently by real-time quantitative PCR (qRT-PCR) for some of the genes and plotted (Additional file 4). There was a high correlation (best-fit linear regression, R^2 ^= 0.76; slope of the regression line, m = 0.91) between the log_2_-transformed values for the microarray and qRT-PCR. The microarray expression assays and their corresponding GEO accession numbers are:

GSM339808: non-light stimulated vs. 2 hr light stimulated rep 1GSM339819: non-light stimulated vs. 2 hr light stimulated rep 2GSM339907: non-light stimulated vs. 2 hr light stimulated rep 3GSM339909: non-light stimulated vs. 2 min light stimulated at -30 min rep 1GSM339910: non-light stimulated vs. 2 min light stimulated at -30 min rep 2GSM339911: non-light stimulated vs. 2 min light stimulated at -30 min rep 3GSM339912: non-light stimulated female head vs. 2 min light stimulated female head at -30 min rep 1GSM339913: non-light stimulated female head vs. 2 min light stimulated female head at -30 min rep 2GSM339914: non-light stimulated female head vs. 2 min light stimulated female head at -30 min rep 3GSM339915: 2 min light stimulated male head at -30 min vs. 2 min light stimulated female head at -30 min rep 1GSM339916: 2 min light stimulated male head at -30 min vs. 2 min light stimulated female head at -30 min rep 2GSM339917: 2 min light stimulated male head at -30 min vs. 2 min light stimulated female head at -30 min rep 3GSM339918: non-light stimulated male head vs. non-light stimulated female head rep 1GSM339943: non-light stimulated male head vs. non-light stimulated female head rep 2GSM339944: non-light stimulated male head vs. non-light stimulated female head rep 3GSM339945: non-light stimulated male head vs. 2 min light stimulated male head at -30 min rep 1GSM339946: non-light stimulated male head vs. 2 min light stimulated male head at -30 min rep 2GSM339947: non-light stimulated male head vs. 2 min light stimulated male head at -30 min rep 3GSM339949: non-bloodfed female head vs. bloodfed female head rep 1GSM339950: non-bloodfed female head vs. bloodfed female head rep 2GSM339951: non-bloodfed female head vs. bloodfed female head rep 3GSM339953: non-bloodfed female carcass vs. bloodfed female carcass rep 1GSM339954: non-bloodfed female carcass vs. bloodfed female carcass rep 2GSM339955: non-bloodfed female carcass vs. bloodfed female carcass rep 3

## Abbreviations

GFP: Green fluorescent protein; TIM: Timeless; PER: Period; CRY: Cryptochrome; CYC: Cycle; CLK: Clock; TO: Takeout; OBP: Odorant binding protein; CCCP: Circadian clock controlled precursor; CK: Casein kinase; PBP/GOBP: Pheromone binding protein/General Odorant binding protein; DD: Dark-Dark; LD: Light-Dark; CT: Circadian time; CIR: Circadian; CSR: Chemosensory; qRT-PCR: Quantative Real-Time PCR; ZT: Zeitgeber time.

## Authors' contributions

SD designed and conducted all the assays and contributed to the writing of the manuscript. GD designed the experiments and the microarrays and contributed to the writing of the manuscript.

## Supplementary Material

Additional file 1 Light-pulse induced alteration in A. gambiae blood feeding.  A. Mosquitoes were exposed to light pulses (of ~800 to 1,000 lux) for 2 min at 120, 90, 60, and 30 min prior to blood provision and light onset (ZT 0). Mosquitoes were allowed to feed for 10 min and were then assigned to one of three categories: fully fed, partially fed or non-fed. The percentage of mosquitoes in each category is shown in the graph; error bars indicate the standard error. Among the controls were the one set that was not exposed to light (continuous dark) and another set that was exposed to continuous light for these 120 min prior to blood provision. B. Mosquitoes were exposed to ~800- to 1,000-lux light pulses for 5, 15, 30, 60, or 120 sec at 30 min prior to light onset and blood provision (ZT 0) for 10 min, after which the numbers were scored and plotted. Significant data points with respect to continuous darkness control set are marked with an asterisk. Click here for file

Additional file 2 Light-pulse induced alteration in A. gambiae blood feeding.  The percentage of non-fed, partially fed and fully fed mosquitoes in all the different light treatments are shown. The standard error and the p-values (t-test) are also shown. Click here for file

Additional file 3 The influence of blood gustatory, temperature and color parameters on the mosquito blood-feeding behavior.  A. Mosquitoes were allowed to probe and feed for 10 min on either 37oC blood (warm blood), 24oC blood (cold blood), 37oC cell line MEM medium (warm red solution), or 37oC water through artificial membrane feeders. The number of mosquitoes probing the membrane was counted every 1 min for 10 min and plotted. The error bars indicate the standard error for six replicate assays. B. The total number (calculated by adding the numbers for each individual minute for 10 min) of mosquitoes that fed on 37oC blood, 24oC blood (cold blood), 37oC MEM medium, or water. Click here for file

Additional file 4 Relationship between the light pulse and blood feeding regulated A. gambiae transcriptome.  A. The experimental design for the light pulse and blood feeding regulated transcriptome studies. The assays were subdivided into two categories: light-pulse treatment (with further subdivision of total mosquitoes and head samples) and blood feeding (further subdivided to head samples and the remaining body). In the light pulse category, six different assays were done (labeled as "a" to "f") and in the blood-fed category, two assays were done (labeled as "g" and "h") and the details of the experimental and control samples/conditions are described. For each assay, the arrowhead points toward the experimental sample (Cy3 label) and the other side refers to the control sample (Cy5 label). B. Pie chart showing the total number of genes (in brackets) that were regulated by both light pulse and blood feeding in different functional groups [CIR: circadian; CSR: chemosensory; PROT/DIG: proteolytic digestion; IMM: immunity; MET: metabolism; CS: cytoskeletal and structural; RED/STR: redox/stress; R/T/T: replication/transcription and translation; TRP: transport]. C. Validation of microarray gene expression data by qRT-PCR. The log2 transformed values generated by microarrays and qRT-PCR were plotted and showed a significant co-relation between the two assays. Click here for file

Additional file 5 The light pulse and blood feeding regulated A. gambiae transcriptome.  List of genes regulated by both light-pulse and blood feeding, along with their previous and recent transcript IDs. Note that the recent AGAP-R IDs for some transcripts are missing in ENSEMBL and so they are left blank in column 2. The list includes only those genes that showed a significant (above the threshold of ±0.8; log2 1.75) level of regulation in at least one of the eight assays ("a" to "h"). The non-significant values (if any) of these genes in other assays are also shown. The gene list is sorted according to the functional groups (discussed in Additional file 4 legend). The eight different assay types ("a" to "h") are listed in the first row. Click here for file

Additional file 6 Correlation of the microarray expression data with the qRT-PCR expression data  The log 2 ratios (experimental /control) of the different assay sets ("a" to "h"), from both the microarray (column 5) and qRT-PCR analysis (column 4) are shown. The gene name is given in column 1, the transcript ID in column 2 and the array set to which the values were compared is listed in column 3. Click here for file

Additional file 7 Determination of RNAi knockdown efficiency of the 10 circadian/chemosensory genes in A. gambiae female mosquitoes after dsRNA injections.  RNA was extracted from different body parts of gene silenced mosquitoes and the GFP dsRNA treated control mosquitoes, and the relative mRNA levels were determined for each gene in both samples. The mRNA level in GFP mosquitoes was set to 1.0, and the corresponding % silencing was determined by qRT-PCR. The AgS7 gene was used for normalization. The standard error values are shown. Click here for file

Additional file 8 Blood-feeding propensity of mosquitoes after RNAi gene silencing  Ten circadian/chemosensory genes were individually silenced in mosquitoes, and the mosquitoes were allowed to feed on blood at the light-on stage in the insectary. The percentages of mosquitoes that did not feed (non-fed) were scored and are shown along with their standard error values. The P- values from statistical analysis; T-test and Mann Whitney Test are also shown. Click here for file

Additional file 9 Fold change in expression of 10 circadian genes selected for RNAi assays.  The -fold change in expression of 10 genes (selected for RNAi assays) in different microarray assays (a, b, c, g, and h; referred in Additional file 4), as determined by qRT-PCR and their corresponding values (if any) from the microarray analyses (Additional file 5). Click here for file

Additional file 10 Primers used for making dsRNAs for RNAi gene silencing. The first 20 bases in bold correspond to the T7 polymerase promoter site. The transcript ID numbers (AGAP-RA from ENSEMBL) are also mentioned for each gene. Click here for file

Additional file 11 Primers used for verification of RNAi silencing. The sense primers were newly designed (VERI Sense) and the antisense primers used were the same as those used for making the corresponding dsRNAs. The transcript ID numbers (AGAP-RA from ENSEMBL) are also mentioned for each gene. Click here for file
