# Supplementary figures and images for: Molecular analysis of photic inhibition of blood-feeding in Anopheles gambiae
Source: BMC Physiol. 2008 Dec 16;8:23. doi: 10.1186/1472-6793-8-23 (PMC2646746; doi:10.1186/1472-6793-8-23)

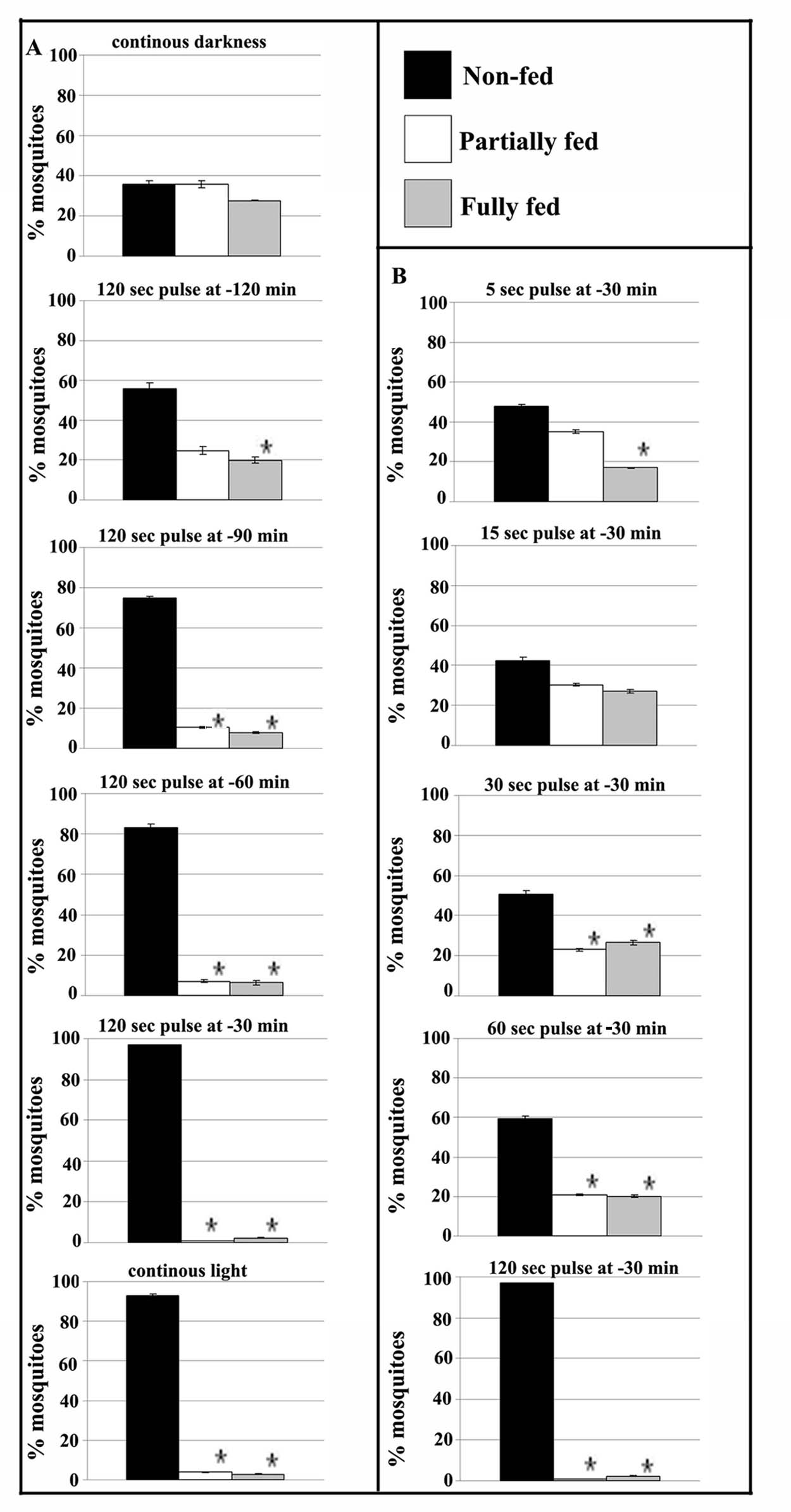

Supplement: Additional file 1 — Light-pulse induced alteration in A. gambiae blood feeding. A. Mosquitoes were exposed to light pulses (of ~800 to 1,000 lux) for 2 min at 120, 90, 60, and 30 min prior to blood provision and light onset (ZT 0). Mosquitoes were allowed to feed for 10 min and were then assigned to one of three categories: fully fed, partially fed or non-fed. The percentage of mosquitoes in each category is shown in the graph; error bars indicate the standard error. Among the controls were the one set that was not exposed to light (continuous dark) and another set that was exposed to continuous light for these 120 min prior to blood provision. B. Mosquitoes were exposed to ~800- to 1,000-lux light pulses for 5, 15, 30, 60, or 120 sec at 30 min prior to light onset and blood provision (ZT 0) for 10 min, after which the numbers were scored and plotted. Significant data points with respect to continuous darkness control set are marked with an asterisk. [file 1472-6793-8-23-S1.jpeg]

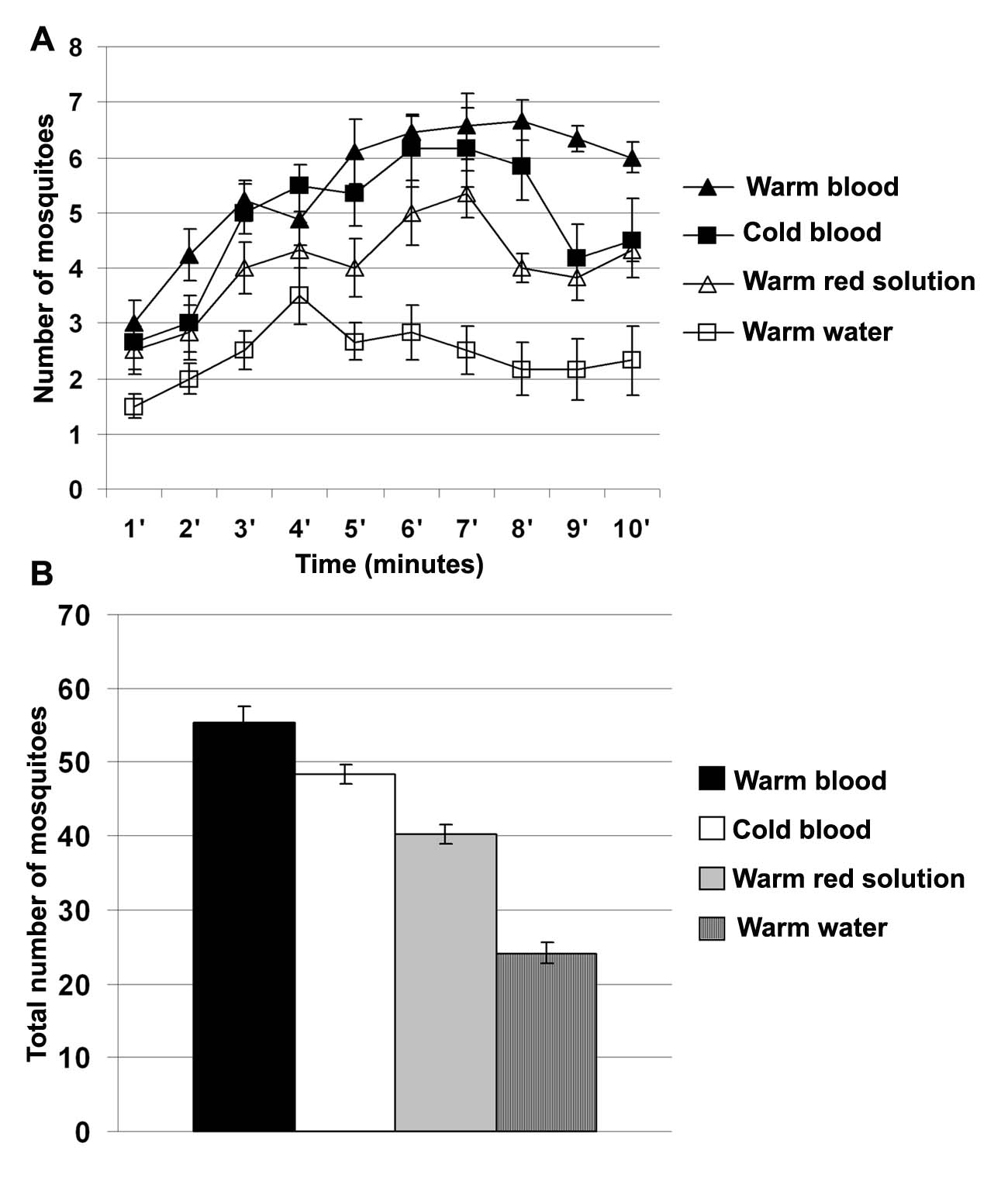

Supplement: Additional file 3 — The influence of blood gustatory, temperature and color parameters on the mosquito blood-feeding behavior. A. Mosquitoes were allowed to probe and feed for 10 min on either 37oC blood (warm blood), 24oC blood (cold blood), 37oC cell line MEM medium (warm red solution), or 37oC water through artificial membrane feeders. The number of mosquitoes probing the membrane was counted every 1 min for 10 min and plotted. The error bars indicate the standard error for six replicate assays. B. The total number (calculated by adding the numbers for each individual minute for 10 min) of mosquitoes that fed on 37oC blood, 24oC blood (cold blood), 37oC MEM medium, or water. [file 1472-6793-8-23-S3.jpeg]

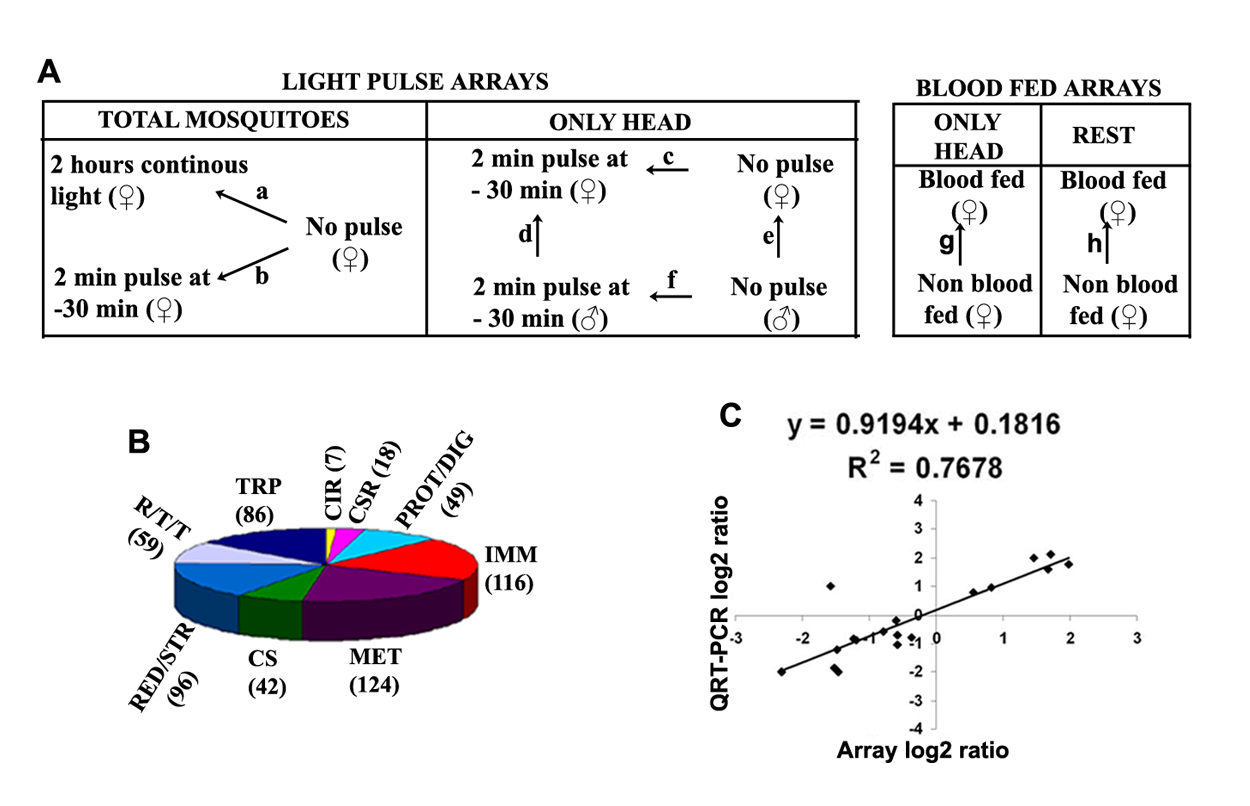

Supplement: Additional file 4 — Relationship between the light pulse and blood feeding regulated A. gambiae transcriptome. A. The experimental design for the light pulse and blood feeding regulated transcriptome studies. The assays were subdivided into two categories: light-pulse treatment (with further subdivision of total mosquitoes and head samples) and blood feeding (further subdivided to head samples and the remaining body). In the light pulse category, six different assays were done (labeled as "a" to "f") and in the blood-fed category, two assays were done (labeled as "g" and "h") and the details of the experimental and control samples/conditions are described. For each assay, the arrowhead points toward the experimental sample (Cy3 label) and the other side refers to the control sample (Cy5 label). B. Pie chart showing the total number of genes (in brackets) that were regulated by both light pulse and blood feeding in different functional groups [CIR: circadian; CSR: chemosensory; PROT/DIG: proteolytic digestion; IMM: immunity; MET: metabolism; CS: cytoskeletal and structural; RED/STR: redox/stress; R/T/T: replication/transcription and translation; TRP: transport]. C. Validation of microarray gene expression data by qRT-PCR. The log2 transformed values generated by microarrays and qRT-PCR were plotted and showed a significant co-relation between the two assays. [file 1472-6793-8-23-S4.jpeg]
