# Supplementary material for: Molecular analysis of photic inhibition of blood-feeding in Anopheles gambiae
Source: BMC Physiol. 2008 Dec 16;8:23. doi: 10.1186/1472-6793-8-23 (PMC2646746; doi:10.1186/1472-6793-8-23)
Supplement: Additional file 2 — Light-pulse induced alteration in A. gambiae blood feeding. The percentage of non-fed, partially fed and fully fed mosquitoes in all the different light treatments are shown. The standard error and the p-values (t-test) are also shown. [file 1472-6793-8-23-S2.doc]

**Additional file 2:**

**Molecular analysis of photic inhibition of blood-sucking behavior in *Anopheles gambiae***

**Suchismita Das^1^ and George Dimopoulos^1, #^**

W. Harry Feinstone Department of Molecular Microbiology and Immunology, Bloomberg School of Public Health, Johns Hopkins University, 615N. Wolfe Street, Baltimore, MD 21205-2179, USA.

# Corresponding author: George Dimopoulos

Email addresses:

SD: [sudas@jhsph.edu](mailto:sudas@jhsph.edu)

GD: [gdimopou@jhsph.edu](mailto:gdimopou@jhsph.edu)

**Additional file 2:**

**Light-pulse induced alteration in *A. gambiae* blood feeding**

The percentage of non-fed, partially fed and fully fed mosquitoes in all the different light treatments are shown. The standard error and the p-values (t-test) are also shown.

| **Light-pulse treatment type** | **Percentage of mosquitoes** | | | **Standard error** | | | **p-value (T-test) of total fed (with respect to continuous darkness for 120 min)** |
| --- | --- | --- | --- | --- | --- | --- | --- |
|  | **Non-fed** | **Partially**  **fed** | **Fully fed** | **Non-fed** | **Partially**  **fed** | **Fully**  **fed** |  |
| Continuous darkness for 120 min | 35.7 | 35.7 | 27.6 | 1.6 | 1.4 | 0.2 | N/A |
| Pulse for 2 min at -120 min | 55.6 | 24.6 | 19.9 | 3.28 | 1.86 | 1.42 | 0.05; Significant |
| Pulse for 2 min at -90 min | 74.6 | 10.3 | 7.95 | 1.13 | 0.59 | 0.54 | <0.001; Significant |
| Pulse for 2 min at -60 min | 83.3 | 7.1 | 6.3 | 1.6 | 0.63 | 0.97 | <0.001: Significant |
| Pulse for 2 min at -30 minutes | 95.9 | 1.3 | 2.8 | 0.24 | 0.10 | 0.14 | <0.001; Significant |
| Continuous light for 120 min | 93 | 3.9 | 2.8 | 0.50 | 0.30 | 0.20 | <0.001; Significant |
| Pulse for 5 seconds at -30 min | 48 | 35 | 17 | 0.96 | 0.73 | 0.23 | 0.06; Non Significant |
| Pulse for 15 seconds at -30 min | 42.5 | 30.3 | 27.2 | 1.51 | 0.56 | 0.95 | 0.20; Non Significant |
| Pulse for 30 seconds at -30 min | 50.5 | 23 | 26.5 | 1.77 | 0.76 | 1.01 | 0.06; Non Significant |
| Pulse for 60 seconds at -30 min | 59.2 | 20.8 | 20 | 1.31 | 0.61 | 0.70 | 0.002; Significant |
