# Supplementary material for: Molecular analysis of photic inhibition of blood-feeding in Anopheles gambiae
Source: BMC Physiol. 2008 Dec 16;8:23. doi: 10.1186/1472-6793-8-23 (PMC2646746; doi:10.1186/1472-6793-8-23)
Supplement: Additional file 6 — Correlation of the microarray expression data with the qRT-PCR expression data The log 2 ratios (experimental /control) of the different assay sets ("a" to "h"), from both the microarray (column 5) and qRT-PCR analysis (column 4) are shown. The gene name is given in column 1, the transcript ID in column 2 and the array set to which the values were compared is listed in column 3. [file 1472-6793-8-23-S6.doc]

**Additional file 6**

**Molecular analysis of photic inhibition of blood-sucking behavior in *Anopheles gambiae***

**Suchismita Das^1^ and George Dimopoulos^1, #^**

W. Harry Feinstone Department of Molecular Microbiology and Immunology, Bloomberg School of Public Health, Johns Hopkins University, 615N. Wolfe Street, Baltimore, MD 21205-2179, USA.

# Corresponding author: George Dimopoulos

Email addresses:

SD: [sudas@jhsph.edu](mailto:sudas@jhsph.edu)

GD: [gdimopou@jhsph.edu](mailto:gdimopou@jhsph.edu)

**Additional file 6:**

**Correlation of the microarray expression data with the qRT-PCR expression data**

The log _2_ ratios (experimental /control) of the different assay sets (“a” to “h”), from both the microarray (column 5) and qRT-PCR analysis (column 4) are shown. The gene name is given in column 1, the transcript ID in column 2 and the array set to which the values were compared is listed in column 3.

| **Gene name** | **Transcript ID** | **Pulse/feeding treatment (array # from Figure 2A)** | **QRT-PCR**  **log_2_ ratio** | **Microarray**  **log_2_ ratio** |
| --- | --- | --- | --- | --- |
| *Timeless* | AGAP010787-RA | Array “a” | 1.60 | 1.67 |
| *Timeless* | AGAP010787-RA | Array “b” | 1.79 | 1.98 |
| *Putative Takeout 1* | AGAP004263-RA | Array “c” | -0.86 | -1.20 |
| *Putative Takeout 1* | AGAP004263-RA | Array “g’ | -1.03 | -0.58 |
| *Putative Takeout 2* | AGAP012703-RA | Array “c” | -1.98 | -1.47 |
| *Putative Takeout 2* | AGAP012703-RA | Array “h” | 2.01 | 1.47 |
| *Putative Takeout 3* | AGAP004262-RA | Array “c” | -0.18 | -0.59 |
| *Putative Takeout 3* | AGAP004262-RA | Array “h” | 2.11 | 1.71 |
| *OBP 4* | AGAP010489-RA | Array “d” | 1.01 | -1.58 |
| *OBP 4* | AGAP010489-RA | Array “h” | -0.81 | -0.36 |
| *OBP 22* | AGAP010409-RA | Array “c” | -1.86 | -1.52 |
| *OBP 22* | AGAP010409-RA | Array “e” | -1.22 | -1.49 |
| *OBP 26* | AGAP012321-RA | Array “a” | -0.58 | -0.78 |
| *OBP 26* | AGAP012321-RA | Array “c” | -1.24 | -0.84 |
| *OBP 26* | AGAP012321-RA | Array “d” | -1.98 | -2.31 |
| *OBP 26* | AGAP012321-RA | Array “h” | -0.69 | -0.57 |
| *Casein kinase II* | AGAP011438-RA | Array “a” | 0.97 | 0.83 |
| *Casein kinase II* | AGAP011438-RA | Array “b” | 0.78 | 0.56 |
