# Supplementary material for: Molecular analysis of photic inhibition of blood-feeding in Anopheles gambiae
Source: BMC Physiol. 2008 Dec 16;8:23. doi: 10.1186/1472-6793-8-23 (PMC2646746; doi:10.1186/1472-6793-8-23)
Supplement: Additional file 7 — Determination of RNAi knockdown efficiency of the 10 circadian/chemosensory genes in A. gambiae female mosquitoes after dsRNA injections. RNA was extracted from different body parts of gene silenced mosquitoes and the GFP dsRNA treated control mosquitoes, and the relative mRNA levels were determined for each gene in both samples. The mRNA level in GFP mosquitoes was set to 1.0, and the corresponding % silencing was determined by qRT-PCR. The AgS7 gene was used for normalization. The standard error values are shown. [file 1472-6793-8-23-S7.doc]

**Additional file 7**

**Molecular analysis of photic inhibition of blood-sucking behavior in *Anopheles gambiae***

**Suchismita Das^1^ and George Dimopoulos^1, #^**

W. Harry Feinstone Department of Molecular Microbiology and Immunology, Bloomberg School of Public Health, Johns Hopkins University, 615N. Wolfe Street, Baltimore, MD 21205-2179, USA.

# Corresponding author: George Dimopoulos

Email addresses:

SD: [sudas@jhsph.edu](mailto:sudas@jhsph.edu)

GD: [gdimopou@jhsph.edu](mailto:gdimopou@jhsph.edu)

**Additional file 7:**

**Determination of RNAi knockdown efficiency of the 10 circadian/chemosensory genes in *A. gambiae* female mosquitoes after dsRNA injections**

RNA was extracted from different body parts of gene silenced mosquitoes and the GFP dsRNA treated control mosquitoes, and the relative mRNA levels were determined for each gene in both samples. The mRNA level in GFP mosquitoes was set to 1.0, and the corresponding % silencing was determined by qRT-PCR. The AgS7 gene was used for normalization. The standard error values are shown.

| **Transcript ID** | **Gene name** | **Tissue type** | **% of silencing**  **with respect to**  **GFP control** | **Standard**  **error** |
| --- | --- | --- | --- | --- |
| AGAP010787-RA | *Timeless* | Antennae | 0.01 | 0.87 |
|  |  | Head | 0 | 1.4 |
|  |  | Rest | 61 | 2.7 |
| **AGAP001856-RA** | *Period* | Antennae | 10 | 1.6 |
|  |  | Head | 20 | 0.68 |
|  |  | Rest | 50 | 1.2 |
| AGAP005711-RA | *Clock* | Antennae | 5 | 2.2 |
|  |  | Head | 20 | 1.7 |
|  |  | Rest | 10 | 1.1 |
| **AGAP001958-RA** | *Cryptochrome 1* | Antennae | 0 | 1.8 |
|  |  | Head | 10 | 0.98 |
|  |  | Rest | 70 | 1.56 |
| AGAP004263-RA | *Putative Takeout 1* | Antennae | 10 | 2.01 |
|  |  | Head | 35 | 1.2 |
|  |  | Rest | 90 | 0.99 |
| AGAP012703-RA | *Putative Takeout 2* | Antennae | 8 | 1.38 |
|  |  | Head | 25 | 1.98 |
|  |  | Rest | 75 | 2.1 |
| AGAP004262-RA | *Putative Takeout 3* | Antennae | 12 | 2.2 |
|  |  | Head | 13 | 1.2 |
|  |  | Rest | 70 | 1.75 |
| AGAP010489-RA | *OBP 4* | Antennae | 20 | 1.1 |
|  |  | Head | 10 | 1.35 |
|  |  | Rest | NA | NA |
| AGAP010409-RA | *OBP 22* | Antennae | 11 | 1.66 |
|  |  | Head | 20 | 1.21 |
|  |  | Rest | NA | NA |
| AGAP012321-RA | *OBP 26* | Antennae | 10 | 1.23 |
|  |  | Head | 10 | 0.87 |
|  |  | Rest | NA | NA |
