# Supplementary material for: Molecular analysis of photic inhibition of blood-feeding in Anopheles gambiae
Source: BMC Physiol. 2008 Dec 16;8:23. doi: 10.1186/1472-6793-8-23 (PMC2646746; doi:10.1186/1472-6793-8-23)
Supplement: Additional file 8 — Blood-feeding propensity of mosquitoes after RNAi gene silencing Ten circadian/chemosensory genes were individually silenced in mosquitoes, and the mosquitoes were allowed to feed on blood at the light-on stage in the insectary. The percentages of mosquitoes that did not feed (non-fed) were scored and are shown along with their standard error values. The P- values from statistical analysis; T-test and Mann Whitney Test are also shown. [file 1472-6793-8-23-S8.doc]

**Additional file 8**

**Molecular analysis of photic inhibition of blood-sucking behavior in *Anopheles gambiae***

**Suchismita Das^1^ and George Dimopoulos^1, #^**

W. Harry Feinstone Department of Molecular Microbiology and Immunology, Bloomberg School of Public Health, Johns Hopkins University, 615N. Wolfe Street, Baltimore, MD 21205-2179, USA.

# Corresponding author: George Dimopoulos

Email addresses:

SD: [sudas@jhsph.edu](mailto:sudas@jhsph.edu)

GD: [gdimopou@jhsph.edu](mailto:gdimopou@jhsph.edu)

**Additional file 8:**

**Blood-feeding propensity of mosquitoes after RNAi gene silencing**

Ten circadian/chemosensory genes were individually silenced in mosquitoes, and the mosquitoes were allowed to feed on blood at the light-on stage in the insectary. The percentages of mosquitoes that did not feed (non-fed) were scored and are shown along with their standard error values. The P- values from statistical analysis; T-test and Mann Whitney Test are also shown.

| **Gene name** | **Percentage of “Non-fed” mosquitoes** | **Standard**  **error** | **p- value**  **(t-test)** | **p-value**  **(Mann**  **Whitney test)** | **Significant (S) /**  **Non-significant**  **(NS)** |
| --- | --- | --- | --- | --- | --- |
| *Timeless* | 24.1 | 1.21 | 0.08 | 0.027 | S |
| *Period* | 35 | 1.02 | 0.71 | 0.4 | NS |
| *Clock* | 28.3 | 1.23 | 0.16 | 0.11 | NS |
| *Cryptochrome 1* | 30 | 1.16 | 0.02 | 0.15 | S |
| *Putative Takeout 1* | 20.8 | 1.25 | 0.04 | 0.04 | S |
| *Putative Takeout 2* | 23.6 | 1.13 | 0.02 | 0.01 | S |
| *Putative Takeout 3* | 18.3 | 1.01 | 0.007 | 0.01 | S |
| *OBP 4* | 57.5 | 1.24 | 0.005 | 0.006 | S |
| *OBP 22* | 44 | 0.99 | 0.2 | 0.19 | NS |
| *OBP 26* | 48.5 | 1.23 | 0.023 | 0.085 | NS |
| *GFP* | 37.5 | 1.19 | N/A | N/A | N/A |
