# Supplementary material for: Molecular analysis of photic inhibition of blood-feeding in Anopheles gambiae
Source: BMC Physiol. 2008 Dec 16;8:23. doi: 10.1186/1472-6793-8-23 (PMC2646746; doi:10.1186/1472-6793-8-23)
Supplement: Additional file 9 — Fold change in expression of 10 circadian genes selected for RNAi assays. The -fold change in expression of 10 genes (selected for RNAi assays) in different microarray assays (a, b, c, g, and h; referred in Additional file 4), as determined by qRT-PCR and their corresponding values (if any) from the microarray analyses (Additional file 5). [file 1472-6793-8-23-S9.doc]

**Additional file 9**

**Molecular analysis of photic inhibition of blood-sucking behavior in *Anopheles gambiae***

**Suchismita Das^1^ and George Dimopoulos^1, #^**

W. Harry Feinstone Department of Molecular Microbiology and Immunology, Bloomberg School of Public Health, Johns Hopkins University, 615N. Wolfe Street, Baltimore, MD 21205-2179, USA.

# Corresponding author: George Dimopoulos

Email addresses:

SD: [sudas@jhsph.edu](mailto:sudas@jhsph.edu)

GD: [gdimopou@jhsph.edu](mailto:gdimopou@jhsph.edu)

**Additional file 9:**

**Fold change in expression of 10 circadian genes selected for RNAi assays**

The **-**fold change in expression of 10 genes (selected for RNAi assays) in different microarray assays (a, b, c, g, and h; referred in Additional file 4), as determined by qRT-PCR and their corresponding values (if any) from the microarray analyses (Additional file 5).

| **Gene**  **name** | **2 hrs continous light vs dark -Total (array a)** | | **Pulse at -30 mins vs no pulse –Total (array b)** | | **Pulse at -30 mins vs no pulse –Head (array c)** | | **Blood-fed vs unfed-Head**  **(array g)** | | **Blood-fed vs unfed-Rest**  **(array h)** | |
| --- | --- | --- | --- | --- | --- | --- | --- | --- | --- | --- |
|  | **qRT-PCR** | **Micro-**  **array** | **qRT-PCR** | **Micro-**  **array** | **qRT-PCR** | **Micro-**  **array** | **qRT-PCR** | **Micro-**  **array** | **qRT-PCR** | **Micro-**  **array** |
| *Timeless* | 1.60 | 1.67 | 1.79 | 1.98 | 0.37 | 0.36 | -0.62 | NA | -0.17 | NA |
| *Period* | 0.07 | NA | 0.28 | NA | 0.44 | NA | -0.20 | NA | 0.97 | NA |
| *Clock* | 0.30 | NA | 0.65 | NA | -0.18 | NA | -0.42 | NA | 0.30 | NA |
| *Cryptochrome1* | -0.10 | NA | -0.04 | 0.55 | 0.42 | 0.34 | 0.16 | 0.35 | 0.01 | 0.63 |
| *Takeout1* | 0.45 | NA | 0.75 | NA | -0.86 | -1.2 | -1.03 | -0.58 | 0.49 | NA |
| *Takeout2* | -0.56 | NA | 0.54 | NA | -1.98 | -1.47 | -0.47 | NA | 2.00 | 1.47 |
| *Takeout3* | -0.03 | NA | 0.42 | NA | -0.18 | -0.59 | -0.40 | NA | 2.11 | 1.71 |
| *OBP4* | 0.08 | NA | 0.30 | NA | -1.82 | NA | -0.84 | NA | -0.81 | -0.36 |
| *OBP22* | 1.07 | NA | 0.83 | NA | -1.86 | -1.52 | -0.20 | NA | 0.54 | NA |
| *OBP26* | -0.58 | -0.78 | -0.74 | NA | -1.24 | -0.84 | 0.42 | NA | -0.69 | -0.57 |
