# Supplementary material for: Molecular analysis of photic inhibition of blood-feeding in Anopheles gambiae
Source: BMC Physiol. 2008 Dec 16;8:23. doi: 10.1186/1472-6793-8-23 (PMC2646746; doi:10.1186/1472-6793-8-23)
Supplement: Additional file 10 — Primers used for making dsRNAs for RNAi gene silencing. The first 20 bases in bold correspond to the T7 polymerase promoter site. The transcript ID numbers (AGAP-RA from ENSEMBL) are also mentioned for each gene. [file 1472-6793-8-23-S10.doc]

**Additional file 10**

**Molecular analysis of photic inhibition of blood-sucking behavior in *Anopheles gambiae***

**Suchismita Das^1^ and George Dimopoulos^1, #^**

W. Harry Feinstone Department of Molecular Microbiology and Immunology, Bloomberg School of Public Health, Johns Hopkins University, 615N. Wolfe Street, Baltimore, MD 21205-2179, USA.

# Corresponding author: George Dimopoulos

Email addresses:

SD: [sudas@jhsph.edu](mailto:sudas@jhsph.edu)

GD: [gdimopou@jhsph.edu](mailto:gdimopou@jhsph.edu)

**Additional file 10:**

Primers used for making dsRNAs for RNAi gene silencing. The first 20 bases in bold correspond to the T7 polymerase promoter site. The transcript ID numbers (AGAP-RA from ENSEMBL) are also mentioned for each gene.

1*.* Timeless: AGAP010787-RA

Sense: **TAATACGACTCACTATAGGG**GTACAGTGTCGGCAGAGCAAAT

Antisense: **TAATACGACTCACTATAGGG**TTGACGAAGCAACAGTCCAGCA

2. Period**: AGAP001856-RA**

Sense: **TAATACGACTCACTATAGGG**AGTGGACCAGCTTCGTGAAT

Antisense: **TAATACGACTCACTATAGGG**CGCTCCGAGAACGTAAAGTC

3. Cryptochrome 1: **AGAP001958-RA**

Sense: **TAATACGACTCACTATAGGG**AACATAACCGCGACCTTCCTG

Antisense: **TAATACGACTCACTATAGGG**CGACCATCGGTGCTGGATACT

4. Putative Takeout 1: AGAP004263-RA

Sense: **TAATACGACTCACTATAGGG**TCGTGCAAGCCATAACCAATA

Antisense: **TAATACGACTCACTATAGGG**ATCGCAGTTCACCATTGTCAT

5. Putative Takeout 2: AGAP012703-RA

Sense: **TAATACGACTCACTATAGGG**AGGCGTTAGCGGTGCGTGTAA

Antisense: **TAATACGACTCACTATAGGG**TCATCTCCATCTTGGTAGGAT

6. Putative Takeout 3: AGAP004262-RA

Sense: **TAATACGACTCACTATAGGG**AGTGCATTTACCGTCCAGTG

Antisense: **TAATACGACTCACTATAGGG**CTCCATCTTGGTAGGATTAGA

7. Clock: AGAP005711-RA

Sense: **TAATACGACTCACTATAGGG**ATACTGCTGAACCCGACGAC

Antisense: **TAATACGACTCACTATAGGG**TGAACTCGCTCTTGGTGTTGT

8. OBP 4: AGAP010489-RA

Sense: **TAATACGACTCACTATAGGG**TATCGGTGCTGGTATCGTCT

Antisense: **TAATACGACTCACTATAGGG**ACGGGTCCTTGTATGAGGT

9. OBP 22: AGAP010409-RA

Sense: **TAATACGACTCACTATAGGG**TGGTAGTGCTGAATGTCCA

Antisense: **TAATACGACTCACTATAGGG**TACATTTCATAGTCTTCCCT

10. OBP 26: AGAP012321-RA

Sense: **TAATACGACTCACTATAGGG**ACGCCTCCAACGCCACCATGA

Antisense: **TAATACGACTCACTATAGGG**TCTCGATGACCGTCTTCTCAT
