# Supplementary material for: Molecular analysis of photic inhibition of blood-feeding in Anopheles gambiae
Source: BMC Physiol. 2008 Dec 16;8:23. doi: 10.1186/1472-6793-8-23 (PMC2646746; doi:10.1186/1472-6793-8-23)
Supplement: Additional file 11 — Primers used for verification of RNAi silencing. The sense primers were newly designed (VERI Sense) and the antisense primers used were the same as those used for making the corresponding dsRNAs. The transcript ID numbers (AGAP-RA from ENSEMBL) are also mentioned for each gene. [file 1472-6793-8-23-S11.doc]

**Additional file 11**

**Molecular analysis of photic inhibition of blood-sucking behavior in *Anopheles gambiae***

**Suchismita Das^1^ and George Dimopoulos^1, #^**

W. Harry Feinstone Department of Molecular Microbiology and Immunology, Bloomberg School of Public Health, Johns Hopkins University, 615N. Wolfe Street, Baltimore, MD 21205-2179, USA.

# Corresponding author: George Dimopoulos

Email addresses:

SD: [sudas@jhsph.edu](mailto:sudas@jhsph.edu)

GD: [gdimopou@jhsph.edu](mailto:gdimopou@jhsph.edu)

**Additional file 11:**

Primers used for verification of RNAi silencing. The sense primers were newly designed (VERI Sense) and the antisense primers used were the same as those used for making the corresponding dsRNAs. The transcript ID numbers (AGAP-RA from ENSEMBL) are also mentioned for each gene.

1. Timeless: AGAP010787-RA

VERI Sense: TGACGCTGTCGACCACGCTA

Antisense: **TAATACGACTCACTATAGGG**TTGACGAAGCAACAGTCCAGCA

2. Period: **AGAP001856-RA**

VERI Sense: ACCGGTTTCTGGTGAACAAC

Antisense: **TAATACGACTCACTATAGGG**CGCTCCGAGAACGTAAAGTC

3. Cryptochrome 1: **AGAP001958-RA**

VERI Sense: TGCCTGAACATTCCGTGGTACA

Antisense: **TAATACGACTCACTATAGGG**CGACCATCGGTGCTGGATACT

4. Putative Takeout 1: AGAP004263-RA

VERI Sense: TGGTGGCTTTAGGCAGTGGA

Antisense: **TAATACGACTCACTATAGGG**ATCGCAGTTCACCATTGTCAT

5. Putative Takeout 2: AGAP012703-RA

VERI Sense: TCTCGCGTGTGATCAATCCT

Antisense: **TAATACGACTCACTATAGGG**TCATCTCCATCTTGGTAGGAT

6. Putative Takeout 3: AGAP004262-RA

VERI Sense: TGCGATCGGTTCTGACTAGT

Antisense: **TAATACGACTCACTATAGGG**CTCCATCTTGGTAGGATTAGA

7. Clock: AGAP005711-RA

VERI Sense: AGCCCTCCTTTCTCTCGAAC

Antisense: **TAATACGACTCACTATAGGG**TGAACTCGCTCTTGGTGTTGT

8. OBP 4: AGAP010489-RA

VERI Sense: ACTGCTGGTTAGTGTTGGCT

Antisense: **TAATACGACTCACTATAGGG**ACGGGTCCTTGTATGAGGT

9. OBP 22: AGAP010409-RA

VERI Sense: ACTCCCTTCTTCTCATCGGA

Antisense: **TAATACGACTCACTATAGGG**TACATTTCATAGTCTTCCCT

10. OBP 26: AGAP012321-RA

VERI Sense: CAGCAATCGTCTGATAAGCT

Antisense: **TAATACGACTCACTATAGGG**TCTCGATGACCGTCTTCTCAT
